# NOP2/Sun RNA Methyltransferase 4 Regulates the Mammalian Target of Rapamycin Signaling Pathway to Promote Hepatocellular Carcinoma Progression

**DOI:** 10.5152/tjg.2024.23684

**Published:** 2025-01-01

**Authors:** Congren Wang, Shaoying Ke, Shaoze Lin, Conglin Lin, Zhibing Cai, Lingju Hong, Qunxiong Pan

**Affiliations:** Department of Hepatobiliary Surgery, First Hospital of Quanzhou Affiliated to Fujian Medical University, Quanzhou, China

**Keywords:** NOP2/Sun RNA methyltransferase 4,, mammalian target of rapamycin,, hepatocellular carcinoma,, malignant progression

## Abstract

**Background/Aims:**

NOP2/Sun RNA methyltransferase 4 (NSUN4) is a prognostic indicator for hepatocellular carcinoma (HCC). However, the mechanism of NSUN4 in HCC is still unexplored. This project mainly focuses on the function and mechanism of NSUN4 in HCC malignant progression.

**Materials and Methods:**

The relation between the expression level of NSUN4 and the prognosis of HCC was measured by the means of bioinformatics. The expression level of NSUN4 was assessed by quantitative reverse transcription polymerase chain reaction. The western blot was utilized to determine the protein expression level of NSUN4 and mammalian target of rapamycin (mTOR) pathway-related proteins in cells and mouse tumor tissues. Cell counting kit-8 and colony formation assays were employed to measure cell proliferation ability. The wound healing assay and Transwell experiment were conducted to measure the cells’ migration and invasion abilities. Flow cytometry was applied to determine the cell cycle.

**Results:**

NSUN4 was overexpressed in HCC tissues and cells, enhancing cell migration, proliferation, and invasion. The influence that NSUN4 exerted on HCC malignant progression could be reduced by the inhibitor of the mTOR pathway.

**Conclusion:**

The study explained the mechanism and influence of NSUN4 on HCC progression by regulating the mTOR signaling pathway through in vitro and in vivo experiments, providing the theoretical basis and a new research direction for clinical prognostic prediction and treatment.

**Main Points**

- NSUN4 was overexpressed in HCC, enhancing cell migration, proliferation, and invasion.
- NSUN4 could activate mTOR signaling pathway.
- The study found that NSUN4 regulated the mTOR signaling pathway to promote HCC progression for the first time.

## Introduction

Liver cancer is one of the most prevalent cancers around the world, and hepatocellular carcinoma (HCC) is the primary malignant tumor of hepatic cells. The majority of HCC patients are already in the advanced stage when they are first diagnosed, with a low 5-year survival rate due to the absence of effective treatments for advanced HCC. Therefore, the early diagnosis of HCC is a major challenge at present.^1-3^ Research has uncovered that HCC often comes with genetic and epigenetic aberrations, including histone changes, chromatin remodeling, non-coding RNA expression, and DNA methylation, which are tightly linked with the progression and metastasis of HCC.^4^

RNA methylation is a novel epigenetic modification. Together with its related downstream signaling pathways, RNA methylation is implicated in numerous biological processes such as cell differentiation and stress response.^5^ Since RNA methylation is also linked to cancer progression, it can be used in the evaluation of tumor prognosis and immune microenvironment. NOP2/Sun RNA methyltransferase 4 (NSUN4), as a regulatory factor of 5-methylcytosine (m5C),^6^ is involved in cancer development according to many reports. For example, based on the Kyoto Encyclopedia of Genes and Genomes analysis (KEGG), Cui et al^7^ pointed out that NSUN4 is mainly enriched in RNA degradation, mammalian target of rapamycin (mTOR) signaling pathway, and adherens junction pathway. Furthermore, according to regression analysis combining clinicopathological parameters, the study also showed that NSUN4 is regarded as an independent prognostic factor. Li et al^8^ discovered a similar result in clear cell renal cell carcinoma, indicating that NSUN4 serves as a marker for diagnosis and prognosis. Although NSUN4 is often used as a marker for immunotherapy and prognosis, its molecular mechanism in HCC has not been reported yet. Therefore, it is of great significance to conduct research in this aspect.

As a protein kinase, mTOR modulates cell growth, survival, metabolism, and immunity.^9^ In human cancers, mTOR is often disordered, and its activation can frequently result in an increase in tumor growth and metastasis.^10^ Given its characteristics, mTOR is an object in cancer research. Holloway et al^11^ discovered that inhibiting both mTOR and human epidermal growth factor receptor 2 (HER2) can effectively inhibit glycolysis in breast cancer, thus achieving a better therapeutic effect. Luo et al^12^ found a similar phenomenon in esophageal squamous cell carcinoma (ESCC), where the abnormal activation of the P13K/Akt/mTOR signaling pathway can cause abnormal growth, differentiation, migration, metabolism, and proliferation of ESCC. Zhu et al^13^ reported that ubiquitin conjugating enzyme E2 T (UBE2T) can up-regulate autophagy in human lung adenocarcinoma cells by activating the p53/AMPK/mTOR signaling pathway. In HCC, mTOR is also abnormally activated.^14,15^ mTORC1 can stimulate m6A mRNA methylation through WTAP expression and SAM synthesis.^16^ However, the relationship between this kind of activation and RNA methylation is not fully understood.

The study analyzed the expression of NSUN4 and the influence of NSUN4 on HCC progression, revealing that the expression of NSUN4 in HCC tissues and cells increased remarkably. According to gene set enrichment analysis (GSEA), NSUN4 was enriched in the mTOR pathway, through which the overexpression of NSUN4 could impact on migration, proliferation, invasion, and cycle of HCC cells. In vivo experiments have confirmed that the overexpression of NSUN4 can affect the growth of HCC through the mTOR signaling pathway. In a word, this project explained the molecular mechanism of NSUN4 and extended the function of NSUN4/mTOR in HCC, shedding new light on future HCC research.

## Materials and Methods

### Bioinformatics Analysis

The FPKM data (Normal: 50, Tumor: 374) and clinical information (time.txt, stage.txt) of HCC were obtained from The Cancer Genome Atlas (TCGA) database. The Wilcoxon test was employed to measure the inter-group difference in NSUN4 expression to draw up violin plots. Groups were divided based on the median level of NSUN4. A Kaplan-Meier (KM) survival analysis was conducted using the survival package. GSEA was applied to perform the pathway enrichment analysis of mRNA.

### Cell Culture

Normal human liver cell line LX-2 (CTCC-ZHYC-0296), human HCC cell lines JHH4 (CTCC-001-0806), SNU-182 (CTCC-400-0146), and HEP3B2.1-7 (CTCC-001-0021) cells were purchased from Meisen CTCC, China. LX-2, JHH4, and HEP3B2.1-7 cells were cultivated in DMEM medium with high glucose. SNU-182 cells were grown in RPMI-1640 medium. Both media were supplemented with 1% penicillin-streptomycin (Beyotime, China) and 10% fetal bovine serum (FBS; XP Biomed Ltd, China) and were cultivated under 5% CO_2_ at 37°C. Ethical approval is not required for this study in accordance with local or national guidelines.

### Cell Transfection

oe-NC and oe-NSUN4 were provided by China’s Fenghui Biotechnology, and si-NC and si-NSUN4 were provided by China’s Tsingke. These plasmids were transfected into HCC cells by utilizing UltraFection 3.0 (4A BIOTECH, China). After 48 hours, we collected the cells.

### Quantitative Reverse Transcription Polymerase Chain Reaction

Total RNA was extracted by means of TRIzol (Cwbio, China). The purity and concentration of RNA were measured by Micro Nucleic Acid Test (OD260/OD280). Reverse transcription of cDNA from 2 μg total RNA was performed with the assistance of Hifair® II first Strand cDNA Synthesis Kit (Yeasn, China). Quantification of relative expression of different genes by PCR was performed using UltraSYBR Mixture (Cwbio, China). The results were analyzed by 2^−^^−ΔΔCt^ to evaluate the relative expression levels of genes. We chose GAPDH as the reference gene. Table 1 is a list of primers.

### Western Blot

The total proteins of each group were extracted from cell lysates, and concentrations of protein were measured by BCA protein assay. Protein samples, isolated by PAGE electrophoresis assay from 20 μg of total proteins, were transferred onto polyvinylidene fluoride (PVDF) membranes. Primary antibodies anti-NSUN4 (ABclonal, China), mTOR (ABclonal, China), p-mTOR (ABclonal, China), p-S6^Ser235/236^ (ABclonal, China), and GAPDH (huabio, China) were used for incubation overnight at 4°C, followed by 2 hours of incubation with a secondary antibody labeled by horseradish peroxidase. Development was formed on a Chemiluminescence Imaging System using a hypersensitive chemiluminescence reagent. ImageJ was utilized to measure the gray value of protein bands. After comparison to GAPDH, the relative expression levels of target proteins were obtained.

### Cell Counting Kit-8

On a 96-well plate, we seeded cells at a density of 2 × 10^3^ cells/well with 100 μL of medium. We analyzed the proliferation of cells by CCK-8 after 0, 24, 48, 72, and 96 hours, respectively. Each well was supplemented with 10 μL of CCK-8 reagent at specific time points. After 2 hours of incubation at 37°C in the dark, we detected the absorbance of each well at a wavelength of 450 nm and drew up the growth curve of each cell group to analyze the proliferation of cells.

### Colony Formation Assay

Every 2 × 10^2^ cells were seeded into a well of a 12-well plate for 5-10 days and immobilized in 75% methanol for 30 minutes at 37°C. Furthermore, we removed the methanol, rinsed the colonies with phosphate-buffered saline (PBS) twice, and stained them in crystal violet for 30 min at 37°C. Finally, we rinsed away the redundant staining agent and took pictures to calculate the number of clones in each well.

### Wound Healing Assay

Every 1 × 10^5^ cells were seeded into a well of a 12-well plate. After the cells attached to the wall, each well was wounded with a sterile pipette tip (200 µL) to draw a cross at the bottom. Phosphate-buffered saline was utilized to rinse the cells, and the wounds were recorded by microscope pictures after 0 and 24 hours, respectively. According to the width of wounds, ImageJ was utilized to measure the migration of cells.

### Transwell

At a density of 1 × 10^4^ cells/well, we seeded cells into Transwell chambers and suspended them in 200 μL of serum-free medium. The invasion assay was conducted in chambers with matrigel. We added DMEM containing 10% FBS in the lower chambers and removed the medium after 24 hours (migration) and 72 hours (invasion). We wiped off redundant medium, matrigel, and cells by using a cotton swab, rinsed cells twice with PBS, and immobolized them in 75% methanol for 30 minutes at 37°C. After 2 times of rinses with PBS, cells were stained with crystal violet for 20 minutes at 37°C in dark areas. After another 2 rinses with PBS, we rinsed the cells with the distilled water twice. We soaked up the liquid of chambers and dried them upside down at 37°. Using operating knife blade, we cut down the membranes of the chambers and put them on glass slides. With neutral resin added and cover glass placed on samples, we calculated the number of migrated and invaded cells under a microscope.

### Flow Cytometry

At a density of 1 × 10^6^ cells/well, cells were seeded into a 6-well plate. A single-cell suspension was formed after treatment. The supernatant was removed after 5 minutes of centrifugation at 1400 rpm. We collected the sediments after a rinse with PBS. Then, we added 500 μL of 70% pre-cooled methanol to resuspend the cells and pipetted them evenly, followed by an overnight incubation of one night at 4°C. After another 5 minutes of centrifugation at 1400 rpm, we removed the supernatant, collected the sediments, and added 500 μL of propidium iodide/ribonuclease stain to incubate for 15 minutes in dark areas. Finally, we analyzed cell cycles and cell arrests in each group using FlowJo-V10 analysis software on a BD Accuri™ C6 Plus (BD Biosciences, USA).

### Construction of Xenograft Tumor Models

Nine BALB/6 female nude mice (15-20 g) around 6 weeks of age were purchased from the Chinese Academy of Sciences (Shanghai) and raised under pathogen-free conditions. SNU-182 cells transfected with oe-NC and oe-NSUN4 plasmids, respectively, were injected subcutaneously into the nude mice to construct a xenograft tumor mouse model. When the tumor size of the constructed mice grew to about 100 mm^3^, the mice were randomly divided into 3 groups of 3 mice each. Phosphate-buffered saline was injected into the abdominal cavity of the nude mice in the first and second groups every other day, and an equal amount of the mTOR pathway inhibitor rapamycin (MCE, USA) (2 mg/kg) was injected into the abdominal cavity of the nude mice in the third group. The longest diameter (L) and the longest transverse diameter (W) in the vertical direction of the tumor were measured by vernier caliper every 7 days, and the tumor volume was calculated according to the formula volume = (L × W^2^)/2. The mice were euthanized after the 21st day, and the tumors were removed for weighing and testing. Animal experiments were performed in strict accordance with the requirements of the Animal Ethics Committee of Quanzhou Medical College (approval number: 2024085; date: June 12, 2024).

Tumor tissues from the above 3 groups of mice were collected, chopped, and ground to make cell suspensions for section 2.5 WB experiments. Primary antibodies included anti-NSUN4 (ABclonal, China), mTOR (ABclonal, China), p-mTOR (ABclonal, China), p-S6Ser235/236 (ABclonal, China), and GAPDH (huabio, China), which were used for cell incubation overnight at 4 °C. Cells were then subjected to 2 hours of incubation with a secondary antibody labeled by horseradish peroxidase.

### Statistical Analysis

We processed all statistical analyses using GraphPad Prism 8 software (GraphPad Software, La Jolla, USA). A *T*-test was employed to compare results between 2 groups. One-way analysis of variance (ANOVA) was employed to compare results between 3 groups. The results presented in all graphs are derived from independent experiments. Each experiment was repeated at least 3times. ^*^P < .05, ^**^P < .01, ^***^P < .001, ^****^P < .0001, ^#^*P* < .05, ^##^*P* < .01, ^###^*P* < .001, ^####^*P* < .0001 were considered statistically significant.

## Results

### NOP2/Sun RNA Methyltransferase 4 Is Up-regulated in Hepatocellular Carcinoma and Relates to Poor Prognosis

**Figure 1.**
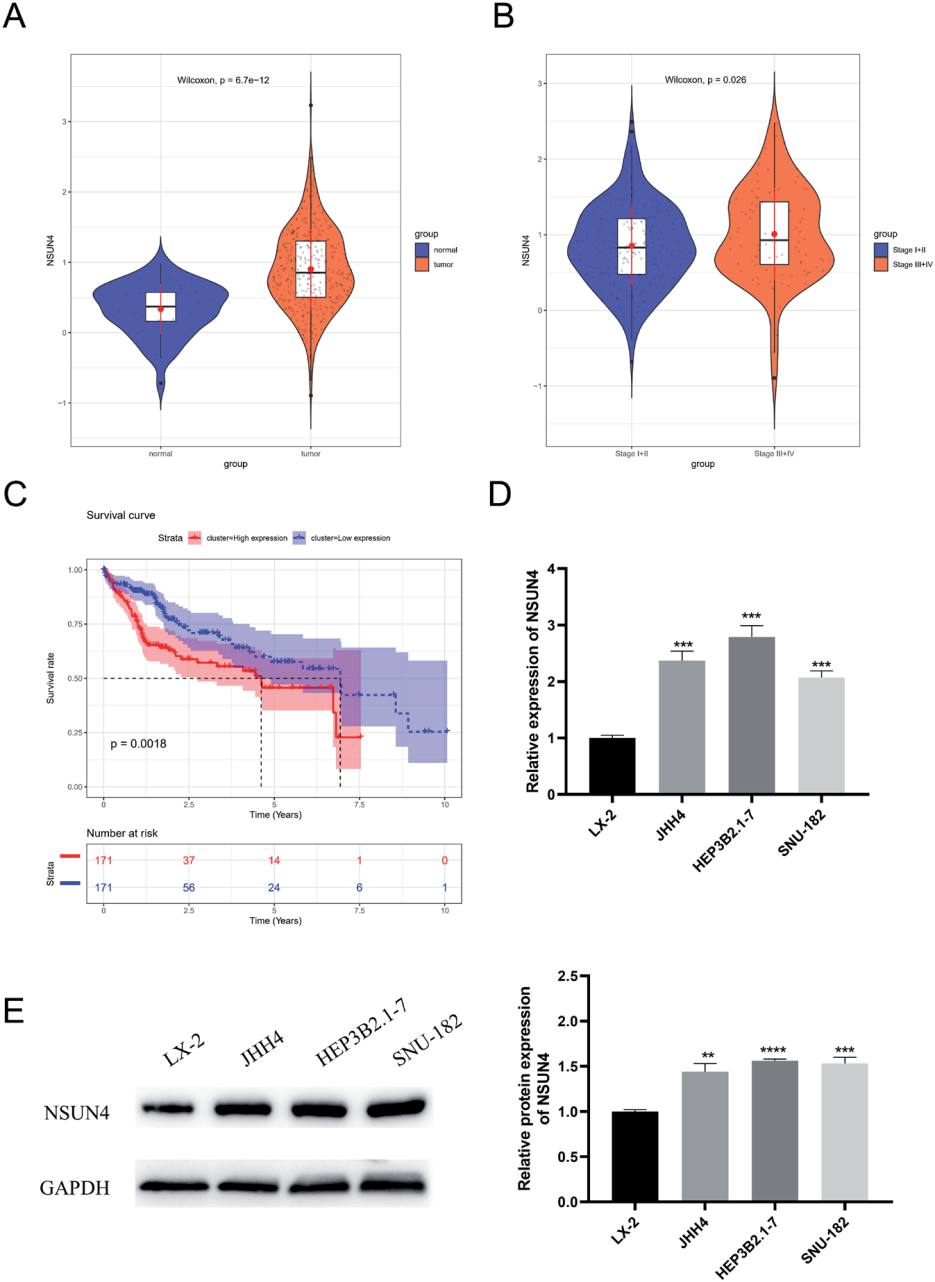
NOP2/Sun RNA methyltransferase 4 is up-regulated in hepatocellular carcinoma and relates to poor prognosis. (A) Bioinformatics analysis of NSUN4 expression in normal tissues and hepatocellular carcinoma tissues. (B) Bioinformatics analysis of NSUN4 expression in tissues of HCC patients with different stages. (C) Bioinformatics analysis of the correlation between NSUN4 expression level and patient prognosis. (D) qRT-PCR detected the levels of NSUN4 in normal human hepatic cells and HCC cells. (E) WB analyzed the levels of NSUN4 in normal human hepatic cells and HCC cells. ^**^P < .01, ^***^P < .001, ^****^P < .0001.

Previous studies have pointed out that NSUN4 is overexpressed in many cancers such as pulmonary squamous carcinoma^6^ and clear cell renal cell carcinoma.^8^ According to bioinformatics analysis, NSUN4 was greatly up-regulated in HCC tissues compared with normal tissues. Additionally, the expression level of NSUN4 in patients in stages III - IV was considerably elevated compared to those in stages I - II. These patients with overexpressed NSUN4 had a poor prognosis and a short average survival time (Figure 1A-C). The qRT-PCR and WB uncovered that compared with the normal human hepatic cell line LX-2, NSUN4 was overexpressed in HCC cells (JHH4, HEP3B2.1-7 and SNU-182) (Figure 1D and E). Among the 3 HCC cells, JHH4, HEP3B2.1-7 and SNU-182, the HEP3B2.1-7 had the highest level of NSUN4 while SNU-182 had the lowest level of NSUN4.

### NOP2/Sun RNA Methyltransferase 4 Facilitates the Malignant Progression of Hepatocellular Carcinoma

**Figure 2.**
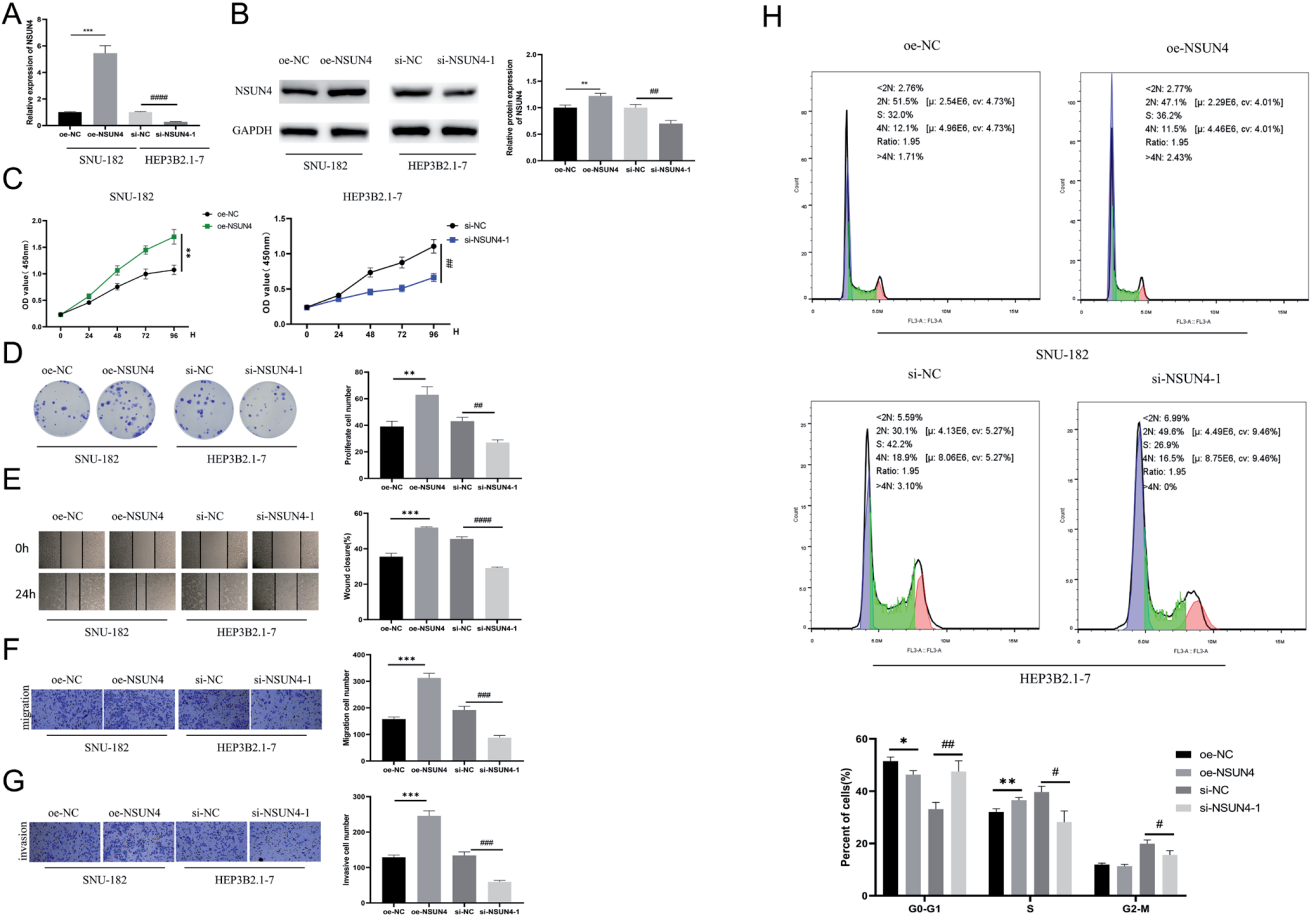
NOP2/Sun RNA methyltransferase 4 facilitates the malignant progression of hepatocellular carcinoma. (A) Quantitative reverse transcription polymerase chain reaction detected the transfection efficiency. (B) WB detected the transfection efficiency. (C) Cell Counting Kit-8 detected the OD value of cells. (D) Colony formation assay assessed the proliferation of cells. (E) Wound healing assay measured the migration of cells. (F) Transwell experiments detected the migration and invasion of cells. (G) Flow cytometry analyzed the cell cycle. The symbol * means the comparison with the oe-NC group and the symbol # means the comparison with the si-NC group. ^*^P < .05, ^**^ *P* < .01, ^***^P < .001, ^#^*P* < .05,^ ##^*P* < .01, ^###^*P* < .001, ^####^*P* < .0001.

**Supplementary Figure 1.**
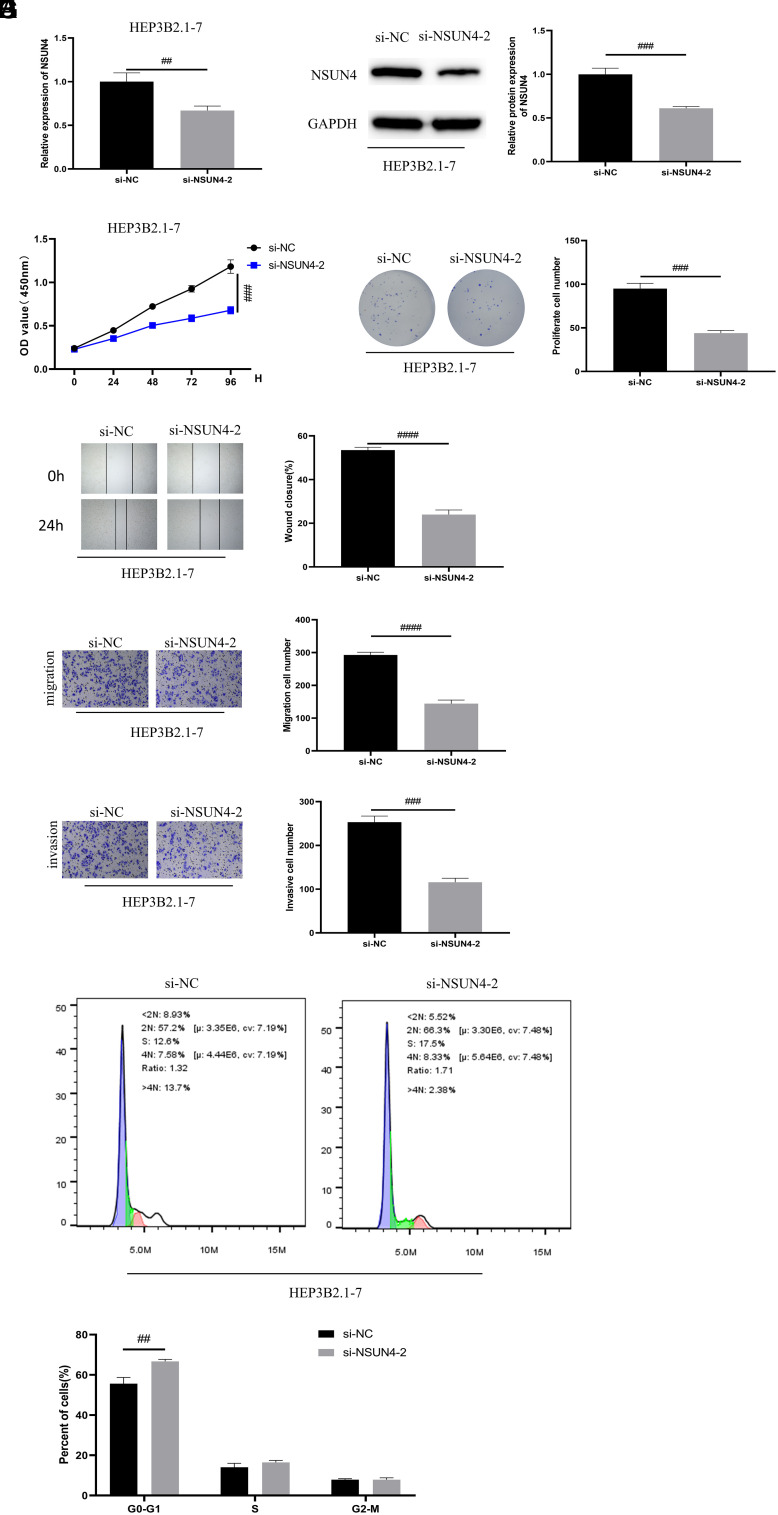
NSUN4 facilitates the malignant progression of HCC. A. qRT-PCR detected the transfection efficiency. B. WB detected the transfection efficiency. C. CCK-8 detected the OD value of cells. D. Colony formation assay assessed the proliferation of cells. E. Wound healing assay measured the migration of cells. F. Transwell experiments detected the migration and invasion of cells. G. Flow cytometry analyzed the cell cycle. The symbol # means the comparison with the si-NC group. ## indicates *P* < 0.01, ### indicates *P* < 0.001, #### indicates *P* < 0.0001.

To elucidate the impact of NSUN4 on HCC malignant progression, we transfected si-NSUN4-1/si-NSUN4-2 into HEP3B2.1-7 and oe-NSUN4 into SNU-182 to establish cell lines with low expression and overexpression of NSUN4. The WB and qRT-PCR results uncovered a transfection efficiency (Figure 2A and B) (Supplementary Figure 1A and B). Additionally, we revealed that the proliferation of HCC cells was effectively repressed effectively after the knockdown of NSUN4, while promoted after the overexpression of NSUN4 (Figure 2C) (Supplementary Figure 1C). Colony formation assay demonstrated that HCC formed fewer colonies after the knockdown of NSUN4, while more after the overexpression of NSUN4 (Figure 2D) (Supplementary Figure 1D). According to wound healing assay and Transwell experiments, a lower expression level of NSUN4 was linked with weaker abilities of cell migration and invasion (Figure 2E-G) (Supplementary Figure 1E-G). A former study demonstrated that the knockdown of NSUN5 can arrest colorectal cancer cells in G0/G1.^17^ In order to illuminate whether NSUN4 can modulate cell cycles similarly, we applied flow cytometry to detect the cell cycle. We found that the knockdown of NSUN4 could greatly cause G0/G1 arrest (Figure 2H) (Supplementary Figure 1H) and the contradictory result was observed when NSUN4 was overexpressed. Taken together, the overexpression of NSUN4 promoted the malignant progression of HCC while repressing NSUN4 suppressed HCC malignant progression.

### NOP2/Sun RNA Methyltransferase 4 Can Activate Mammalian Target of Rapamycin Signaling Pathway

**Figure 3.**
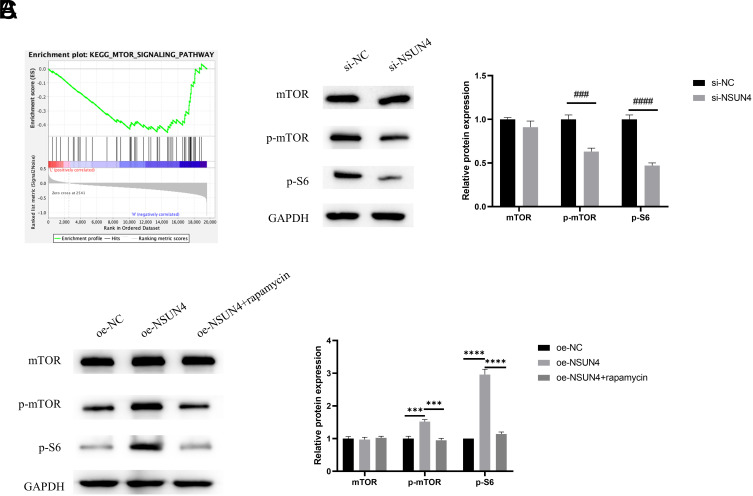
NSUN4 can activate mTOR signaling pathway.(A) GSEA enrichment analysis of NSUN4. (B-C) WB detected the expression levels of mTOR signaling pathway-related proteins (mTOR, p-mTOR, and p-S6^Ser235/236^) in different groups. The symbol * means the comparison with the oe-NC group or the oe-NSUN4 group, the symbol # means the comparison with the si-NC group. ^***^P < .001, ^****^P < .0001,^ ###^*P* < .001, ^####^*P* < .0001.

Conducting a GSEA analysis based on the KEGG database, we observed that NSUN4 was enriched in the mTOR signaling pathway (Figure 3A). We carried out WB to measure the influence of NSUN4 on the mTOR pathway. As results demonstrated, knocking down NSUN4 remarkably decreased the levels of p-mTOR and p-S6^Ser235/236^ (Figure 3B). However, the overexpression of NSUN4 greatly improved levels of p-mTOR and p-S6^Ser235/236^ (Figure 3C). In order to obtain further verification of the relation between NSUN4 and mTOR, we utilized rapamycin, an inhibitor of mTOR, to conduct rescue experiments. We found that, compared with the oe-NSUN4 group, rapamycin inhibited the expression of p-mTOR and p-S6^Ser235/236^ and rescued the influence of NSUN4 overexpression (Figure 3C). Taken together, NSUN4 activated mTOR signaling pathway.

### NOP2/Sun RNA Methyltransferase 4 Triggers Mammalian Target of Rapamycin Signaling Pathway to Promote Hepatocellular Carcinoma Malignant Progression

**Figure 4.**
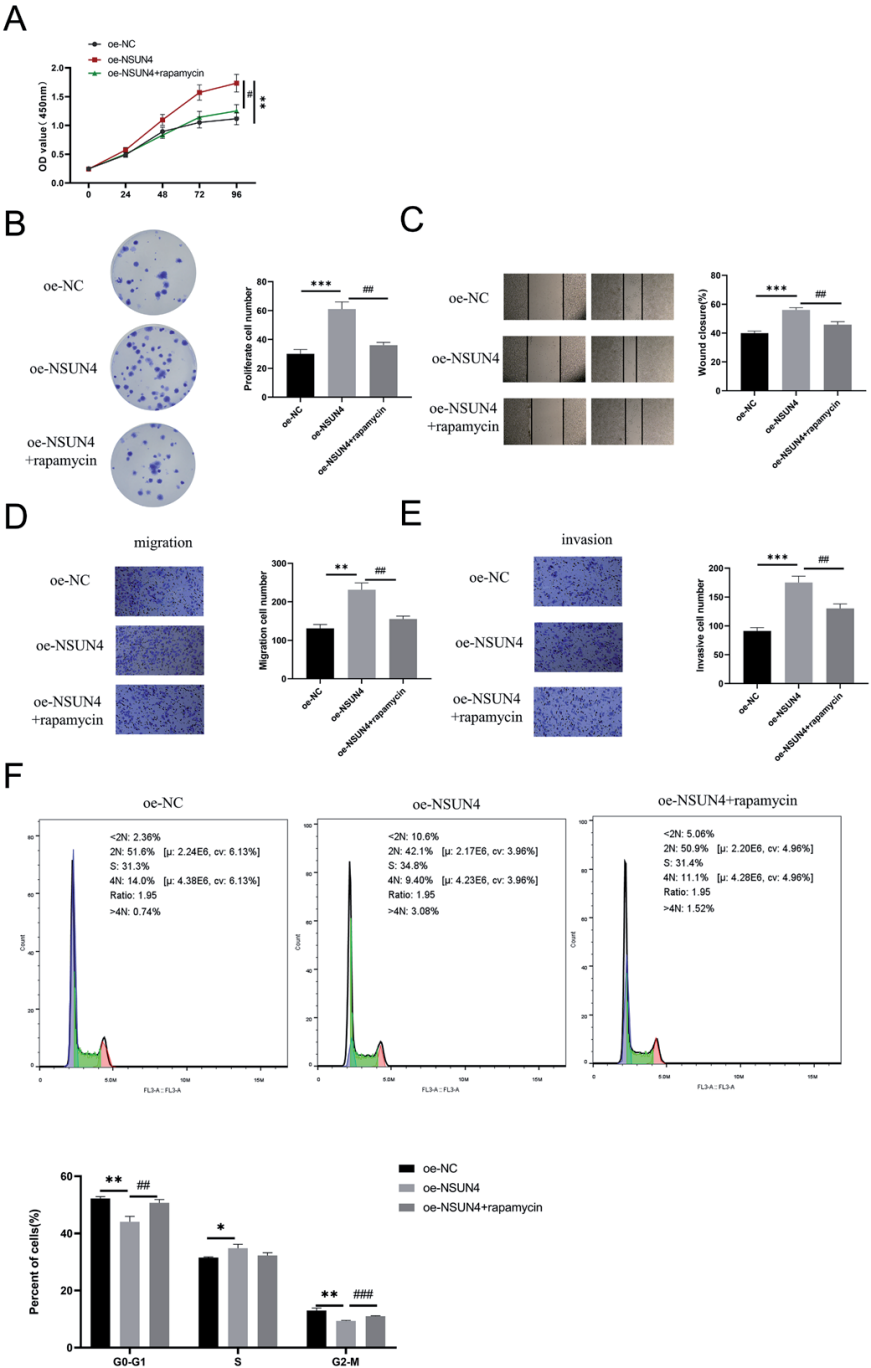
NOP2/Sun RNA methyltransferase 4 triggers the mammalian target of rapamycin signaling pathway to promote hepatocellular carcinoma malignant progression. (A) Cell counting kit-8 measured the proliferation of cells. (B) Colony formation assay detected the proliferation of cells. (C) Wound healing assay analyzed the migration of cells. (D) Transwell experiments tested the migration and invasion of cells. (E) Flow cytometry detected cell cycle. The symbol * means the comparison with the oe-NC group and the symbol # means the comparison with the oe-NSUN4 group. *P < .05, **P < .01, *** *P* < .001, ^#^*P* < .05, ^##^*P* < .01, ^###^*P* < .001.

Having verified that NSUN4 can activate the mTOR signaling pathway, we utilized the CCK-8 experiment and colony formation assay to determine the impact of rapamycin on cell proliferation of cells to further clarify the molecular mechanism of NSUN4 in tumor promotion. The results uncovered that rapamycin effectively reduced tumor promotion caused by NSUN4 overexpression (Figure 4A and B). Furthermore, we tested the influence of rapamycin on migration and invasion of HCC cells by wound healing assay and Transwell experiments. It was revealed that compared with the oe-NSUN4 group, rapamycin repressed the migration and invasion of HCC cells (Figure 4C and E). As flow cytometry showed, cells arrested in G0/G1 reduced remarkably after the overexpression of NSUN4, while the rapamycin treatment caused obvious G0/G1 arrest (Figure 4F). All of these results indicated that NSUN4 activated the mTOR signaling pathway to promote HCC malignant progression.

### Activation of mTOR Signaling Pathway by NOP2/Sun RNA Methyltransferase 4 Promotes Hepatocellular Carcinoma Tumor Progression in Mice in Vivo

**Figure 5.**
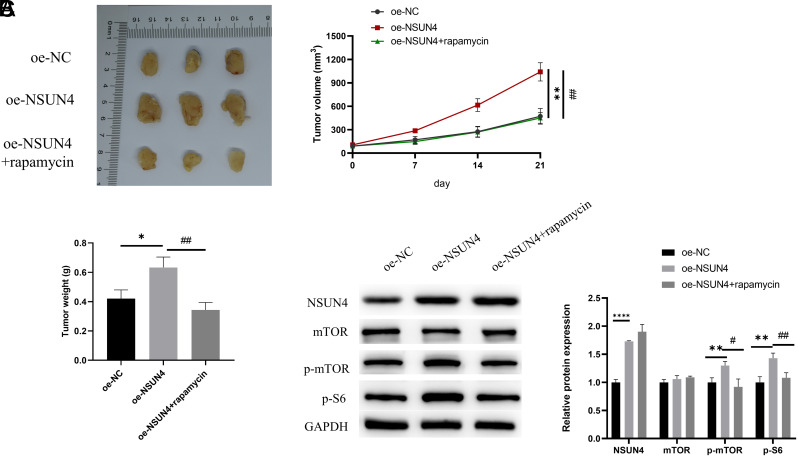
Activation of mammalian target of rapamycin signaling pathway by NSUN4 promotes HCC tumor progression in mice in vivo**. (**A) Volume change curves of different groups of mice. (B). Changes in the mass of mice in different groups. (C) WB detected the expression levels of mTOR signaling pathway-related proteins (NSUN4, mTOR, p-mTOR, and p-S6Ser235/236) in different groups. The symbol * means the comparison with the oe-NC group and the symbol # means the comparison with the oe-NSUN4 group. ^*^P < .05, ^**^P < .01,^ ****^P < .0001, ^#^*P* < .05, ^##^*P* < .01.

The above experimental results indicated that NSUN4 could promote the malignant progression of HCC cells through the activation of the mTOR signaling pathway. In order to investigate whether NSUN4 can promote the growth of HCC tumors through the activation of the mTOR signaling pathway in mice, we constructed the following subgroups based on SNU-182 cells: oe-NC/oe-NSUN4/oe-NSUN4+rapamycin. The results showed that the tumor volume and mass of mice in the oe-NSUN4 group were significantly increased compared to the oe-NC group, whereas rapamycin treatment inhibited tumor growth (Figure 5A and B). The results of the WB experiments showed that the protein expression of NSUN4, p-mTOR, and p-S6^Ser235/236^ was significantly elevated, while there was no effect on the protein expression of NSUN4 after rapamycin treatment, rescuing the effect of overexpression of NSUN4 on the protein expression of p-mTOR, p-S6^Ser235/236^ (Figure 5C). The above experimental results indicated that NSUN4 could promote the growth of HCC tumors by activating the mTOR signaling pathway in mice.

## Discussion

Hepatocellular carcinoma is asymptomatic in its initial stage, often resulting in a late diagnosis. What is worse, HCC is highly resistant to conventional chemotherapy and radiotherapy, and it is frequently accompanied by genetic and epigenetic aberrations.^18^ The methylation level of RNA is regulated by RNA methylation-related modification enzymes and downstream effectors, which exert influence on RNA processing, metabolism, proliferation, migration, and physiological and pathological processes. NOP2/Sun RNA methyltransferase 4 is an important regulator in RNA methylation.^6,19^ Reportedly, the level of RNA methylation is more elevated in HCC cells than in normal cells, and NSUN4 is regarded as the RNA methylation-related gene to predict the prognosis of HCC.^19^ Similarly, He et al^20^ reported that the overexpression of NSUN4 is tightly linked to the survival of HCC patients. In our investigation, through bioinformatics analysis, we first clarified that NSUN4 was overexpressed in HCC, which played a part in HCC patients’ staging and prognosis. Secondly, at the cellular level, we verified that compared with normal cells, NSUN4 was greatly upregulated in HCC cells. Silencing NSUN4 could remarkably reduce the migration, proliferation, and invasion of HCC cells, causing cell cycle arrest in G1 and stopping them from proliferating. That is to say, knockdown of NSUN4 facilitates cells to synthesize RNA and protein and not to enter the next cycle phase. The results of the NSUN4 overexpression group were contrary to those of the si-NSUN4 group. In conclusion, we proved that NSUN4 could promote HCC malignant progression. Apparently, our findings are in line with those of Li et al^19^ who confirmed the high expression of NSUN4 in HCC tissues by IHC experiments.

As an important cell signal of eukaryotes that participates in cell growth, autophagy, and apoptosis and is closely related to tumor development, mTOR is regarded as a new potential target for tumor treatment.^21,22^ For this reason, mTOR is also frequently studied in HCC research. For example, Zhou et al^23^ found that schlafen family member 11 (SLFN11) can inhibit the mTOR signaling pathway in HCC by ribosomal protein S4 X-linked (RPS4X) so as to inhibit the occurrence and metastasis of HCC. SLFN11 was used as a biomarker for prognosis and an inhibitor of tumors. Pu et al^24^ put forward that the interaction of valosin-containing protein (VCP) and high mobility group box 1 (HMGB1) can trigger the PI3K/AKT/mTOR pathway to promote HCC progression. Huang Hui et al^25^ revealed that Osthole can regulate glycolysis of HCC to enhance its radiosensitivity by inhibiting GSK-3β/AMPK/mTOR pathway. However, the relation between NSUN4 and mTOR in HCC has not been elucidated. The relationship between NSUN4 and mTOR is currently only predicted based on bioinformatics methods. For example, Cui et al^7^ confirmed by KEGG analysis that NSUN4 is mainly enriched in signaling pathways involving adhesion molecule junctions, RNA degradation, mTOR signaling pathway, complement, and cohesin cascades. Herein, we uncovered that NSUN4 was enriched in the mTOR pathway by means of bioinformatics analysis. Meanwhile, according to WB, we proved that NSUN4 could activate the mTOR pathway. Then, we verified our results by conducting rescue experiments, finding that the inhibitor of mTOR could effectively rescue the influence of NSUN4 overexpression on HCC malignant progression. In addition, at the cellular level, we revealed that NSUN4 could influence the migration, proliferation, and invasion of HCC by triggering the mTOR pathway. Therefore, the above experimental results confirmed the innovation of our study.

In summary, the project for the first time demonstrated the biological functions of NSUN4 through cell experiments and explained the influence of NSUN4 on HCC malignant progression by targeting mTOR, proffering a theoretical foundation for HCC clinical detection and treatment. However, there are still some limitations in our study. We only demonstrated it at the cellular level, lacking actual clinical studies to support it, and we failed to explore the potential regulatory mechanisms by which NSUN4 targets and affects the mTOR pathway, as well as the potential molecular mechanisms by which mTOR affects HCC, which is our ongoing subject. In a word, our study can shed new light on HCC treatment.

## Supplementary Materials

Supplementary Material

## Figures and Tables

**Table 1.** Primer Sequences in Quantitative Reverse Transcription Polymerase Chain Reaction

Primer	Forward	Reverse
NSUN4	5’-TACCATAGCGACCTTGCCTG-3’	5’-AATTCCAGGGCTTCCAGTGA-3’
GAPDH	5’-ACATCGCTCAGACACCATG-3’	5’-TGTAGTTGAGGTCAATGAAGGG-3’

## Data Availability

The data that support the findings of this study are available upon request from the corresponding author.
