# Supplementary material for: NOP2/Sun RNA Methyltransferase 4 Regulates the Mammalian Target of Rapamycin Signaling Pathway to Promote Hepatocellular Carcinoma Progression
Source: Turk J Gastroenterol. 2025 Jan 1;36(1):24–33. doi: 10.5152/tjg.2024.23684 (PMC11736862; doi:10.5152/tjg.2024.23684)
Supplement: Supplementary Material [file supplementary_material.pdf]

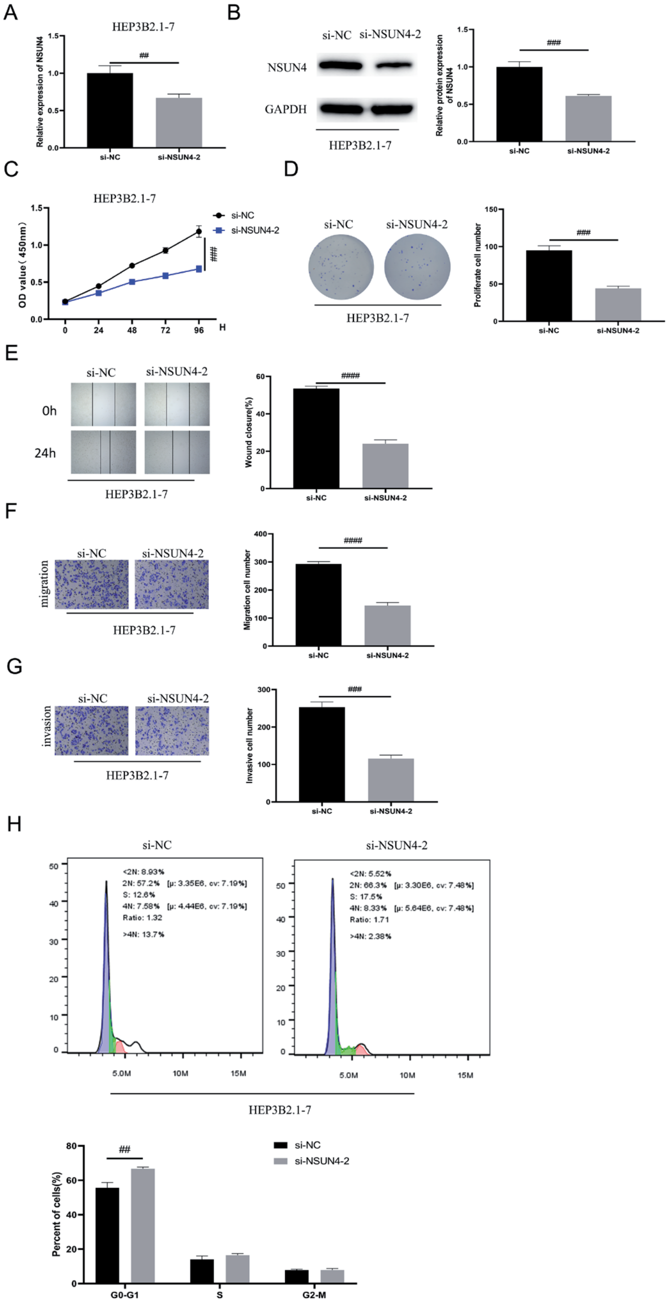

Supplementary Figure 1. NSUN4 facilitates the malignant progression of HCC. A. qRT-PCR detected the transfection efficiency. B. WB detected the transfection efficiency. C. CCK-8 detected the OD value of cells. D. Colony formation assay assessed the proliferation of cells. E. Wound healing assay measured the migration of cells. F. Transwell experiments detected the migration and invasion of cells. G. Flow cytometry analyzed the cell cycle. The symbol # means the comparison with the si-NC group. ## indicates $P < 0.01$, ### indicates $P < 0.001$, #### indicates $P < 0.0001$.
